# TAK1-inhibitors are cytotoxic for multiple myeloma cells alone and in combination with melphalan

**DOI:** 10.18632/oncotarget.28073

**Published:** 2021-10-12

**Authors:** Erling Håland, Ingrid Nyhus Moen, Elias Veidal, Hanne Hella, Kristine Misund, Tobias S. Slørdahl, Kristian K. Starheim

**Affiliations:** ^1^CEMIR Centre of Molecular Inflammation Research, IKOM, NTNU, Trondheim, Norway; ^2^Department of Clinical and Molecular Medicine, NTNU, Trondheim, Norway; ^3^Department of Hematology, St. Olavs University Hospital, Trondheim, Norway

**Keywords:** multiple myeloma, TAK1, apoptosis, NF-κB, cancer treatment

## Abstract

Multiple myeloma (MM) is an incurable cancer caused by malignant transformation of plasma cells. Transforming growth factor-β activated kinase 1 (MAP3K7, TAK1) is a major regulator of nuclear factor kappa-light-chain-enhancer of activated B cells (NF-κB) and mitogen-activated protein kinase (MAPK) signaling. Both NF-κB and MAPK control expression of genes with vital roles for drug resistance in MM. TAK1 is an attractive drug target as it switches these survival pathways to cell death. Our analysis showed that patients with high *MAP3K7* expression in the tumor had shorter overall and progression free survival. The TAK1-inhibitors NG25 and 5Z-7-oxozeaenol (5Z-7) were cytotoxic to MM cell lines and patient cells. NG25 reduced expression of MYC and E2F controlled genes, involved in tumor cell growth, cell cycle progression and drug resilience. TAK1 can be activated by genotoxic stress. NG25 and 5Z-7 induced both synergistic and additive cytotoxicity in combination with the alkylating agent melphalan. Melphalan activated TAK1, NF-κB, and the MAPKs p38 and c-Jun N-terminal kinase (JNK), as well as a transcriptional UV-response. This was blocked by NG25, and instead apoptosis was activated. MM induce elevated bone-degradation resulting in myeloma bone disease (MBD), which is the main cause of disability and morbidity in MM patients. NG25 and 5Z-7 reduced differentiation and viability of human bone degrading osteoclasts, suggesting that TAK1-inhibition can have a double beneficial effect for patients. In sum, TAK1 is a promising drug target for MM treatment.

## INTRODUCTION

Multiple myeloma (MM) is the second most common hematological malignancy worldwide. It is characterized by malignant transformation of clonal plasma cells in the bone marrow accompanied by secretion of monoclonal immunoglobulins, and cancer-induced bone degradation (myeloma bone disease, MBD). The overall survival time of multiple myeloma has improved in the later years due to novel therapeutic strategies [1]. However, the disease is still incurable due to minimal residual disease (MRD), development of drug resistance and relapse. A common treatment strategy in multiple myeloma is induction of DNA damage in the MM cells caused by DNA-damaging agents, such as melphalan. However, they are associated with side effects for the patients and they eventually develop drug resistance [2].

Transforming growth factor-β activated kinase 1 (MAP3K7, TAK1) is a serine/threonine kinase that is activated by a variety of immune receptors, as well as genotoxic stress such as DNA-damage. TAK1 plays an important role in development of chemo resistance in a magnitude of different types of cancer [3]. It induces expression of survival factors through nuclear factor kappa-light-chain-enhancer of activated B cells (NF-κB) and mitogen activated protein kinases (MAPK). MAPK consist of three major families: extracellular-signal-regulated-kinases (ERKs), p38 MAP kinases, and cJun N-terminal kinases (JNKs) [4]. NF-κB and MAPK regulate a wide range of immune- and oncological survival programs, and constitutive NF-κB activation is an important pro-tumor mechanism in MM [5]. Several p38-inhibitors have been tested in clinical trials for several types of cancer, but they have failed due to limited efficiency [6]. Targeting the kinases upstream in the signaling pathway, such as TAK1, has been proposed [7]. TAK1 restrains receptor-interacting serine/threonine-protein kinase 1 (RIPK1) dependent cell death, and inhibition of activated TAK1 shifts cells from pro-survival programs to cell death, making it an attractive drug target [8]. TAK1-inhibition has shown promising results in MM, but with limited investigation [9, 10].

Here, we show that the TAK1-inhibitors NG25 and 5Z-7 reduce viability of MM cell lines and primary cells. The combination of TAK1 inhibitors with melphalan or other DNA-damaging agents increases the cytotoxicity in a synergistic or additive manner. TAK1-inhibitors also reduced number and viability of osteoclasts, suggesting that they have an additional positive effect on MBD. Our findings suggest that TAK1-inhibitors in combination with DNA-damaging agents represent a potential treatment strategy in MM patients.

## RESULTS

### TAK1-inhibitors are cytotoxic to MM cell lines alone and in combination with melphalan

**Figure 1:**
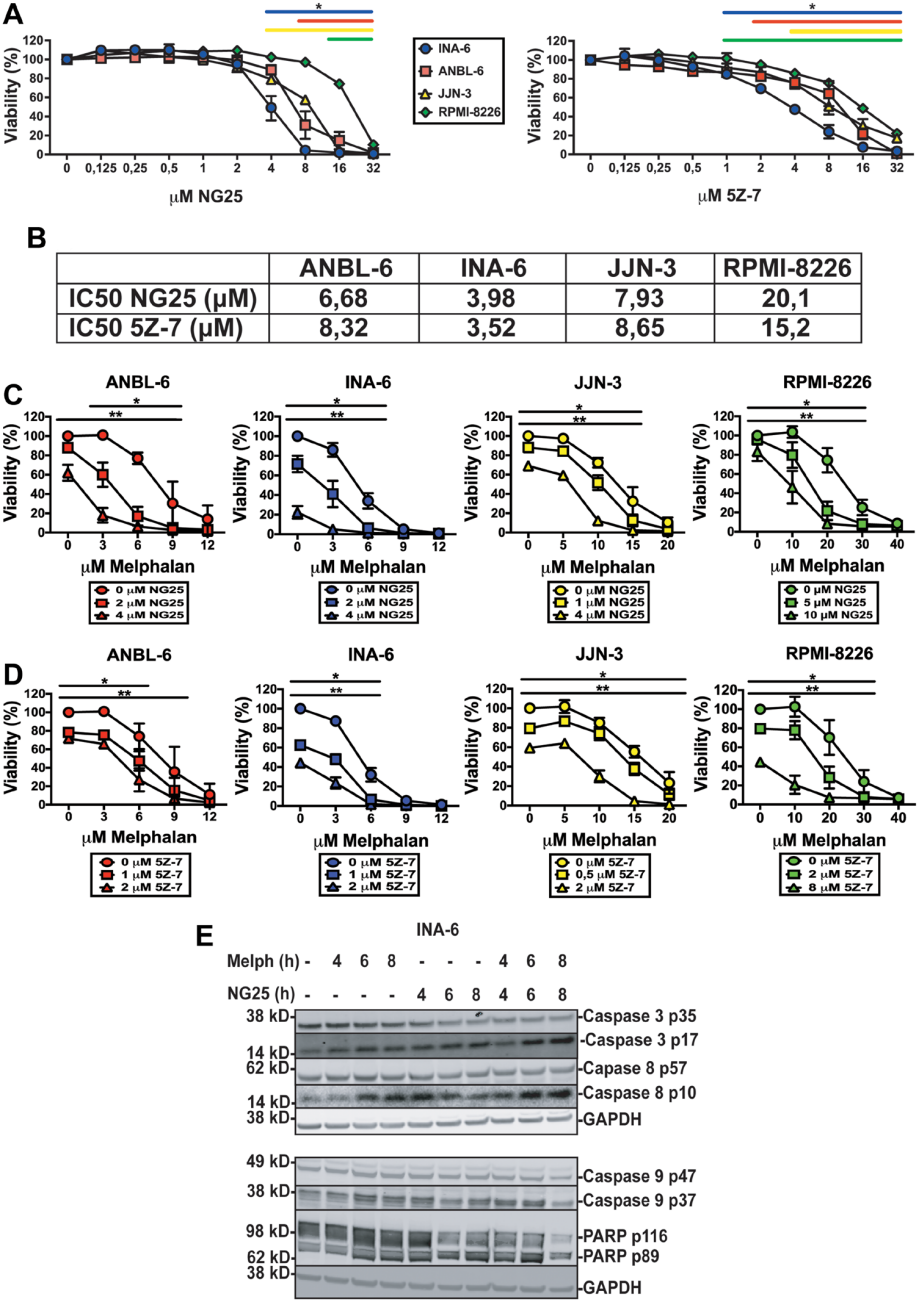
TAK1-inhibitors are cytotoxic to MM cell lines alone and in combination with melphalan. MM cell lines treated with NG25 or 5Z-7 at indicated concentrations for 18 hours before measuring the viability with CellTiter-Glo. (**A**) Mean and SD from three independent experiments are shown. Asterisks indicate statistically significant differences (One-way ANOVA, Tukey’s multiple comparison test, *P* < 0,05) compared with untreated. (**B**) IC50-values of NG25 and 5Z-7 in MM cell lines. (**C**) MM cell lines treated with NG25 in combination with melphalan. Asterisks indicate statistically significant differences (two-way ANOVA, Tukey’s multiple comparison test, *P* < 0,05) compared with the control not treated with NG25. Single asterisk denotes statistical significance as compared to the NG25-untreated for the lowest dose of NG25, double asterisk denotes statistical significance as compared to the untreated control for the highest dose of NG25. (**D**) MM cell lines treated with 5Z-7 in combination with melphalan. Asterisks indicate statistically significant differences (two-way ANOVA, Tukey’s multiple comparison test, *P* < 0,05) compared with the control not treated with 5Z-7. Single asterisk denotes statistical significance as compared to the control not treated with 5Z-7 for the lowest dose of 5Z-7, double asterisk denotes statistical significance as compared to the untreated control for the highest dose of 5Z-7. (**E**) MM cells from the INA-6 cell line were treated with 2 μM NG25 or 10 μM melphalan or both at the indicated time points (hours, h), and cell lysates were analyzed for full length and cleaved caspase 3, caspase 8, caspase 9, and PARP protein levels by immunoblotting. GAPDH is loading control, shown for corresponding membranes. One representative of three independent experiments is shown, and full membranes of all experiments are given in Supplementary Figure 4.

Analysis of data from the MMRF CoMMpass study showed that MM patients with high expression of *MAP3K7* had lower overall and progression free survival (Supplementary Figure 1A and 1B). On the basis of this we tested whether the TAK1-inhibitors NG25 and 5Z-7 affected viability of MM cell lines. Both NG25 and 5Z-7 decreased viability of the cell lines INA-6, ANBL-6, JJN-3, and RPMI-8226 (Figure 1A and 1B). This is in accordance with previous works [10]. Sensitivity of cell lines varied, but IC50 was generally lower than in PBMCs from healthy donors (Supplementary Figure 1C and 1D, Supplementary Table 1).

As TAK1 can activate survival mechanisms after DNA-damage we hypothesized TAK1-inhibitors would render MM-cells more sensitive to melphalan [3]. Both NG25 and 5Z-7 decreased IC50 of melphalan in all tested cell lines (Figure 1C, 1D, Supplementary Table 2). Similar but less pronounced effects were seen in combination with the DNA-damaging drugs doxorubicin and etoposide (Supplementary Figure 1E and 1F and Supplementary Tables 3 and 4).

NG25-treatment induced cleavage of activator caspases 8 and 9, as well as the effector caspase 3 and PARP in INA-6 cells. This was further activated in melphalan + NG25-treated cells (Figure 1E, Supplementary Figure 4). This shows that TAK1-inhibition activates apoptosis, and that this is partly through caspase 8.

### NG25 blocks melphalan-induced p38, ERK and NF-κB signaling

**Figure 2:**
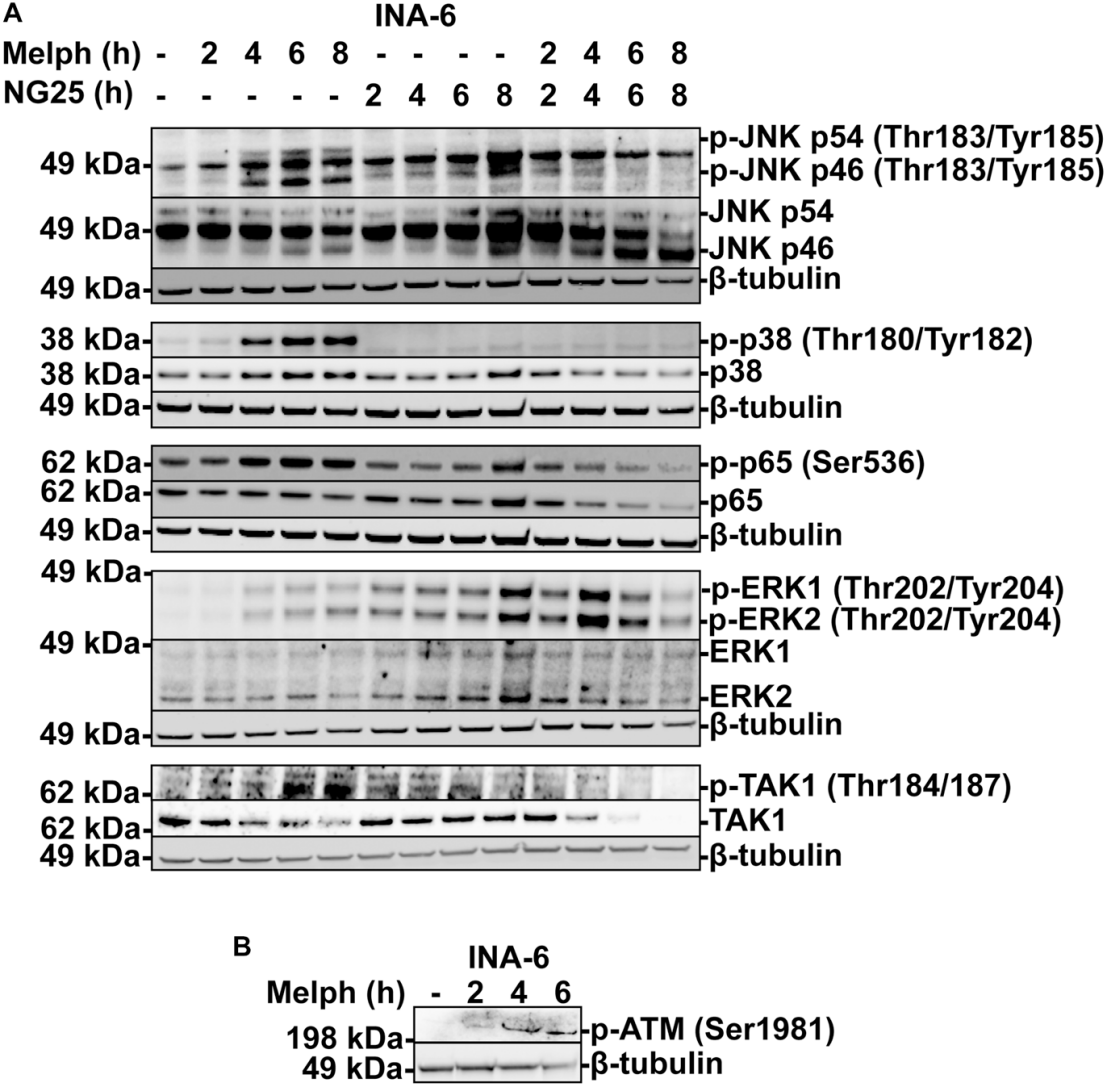
NG25 blocks melphalan-induced p38, ERK- and NF-κB signaling. (**A**) MM cells from the INA-6 cell line were treated with 2 μM NG25 or 10 μM melphalan or both for the indicated time, and cell lysates were analyzed for protein levels of the indicated antigens by immunoblotting. β-Tubulin is loading control, shown for corresponding membranes. One representative of three independent experiments is shown, and full membranes from all experiments are given in Supplementary Figure 5. (**B**) MM cells from the INA-6 cell line were treated with 10 μM melphalan at the indicated time points (hours, h), and cell lysates were analyzed for p-ATM protein levels by immunoblotting. β-Tubulin is loading control. One representative of three independent experiments is shown, and full membranes from all experiments are given in Supplementary Figure 6.

Melphalan is an alkylating agent that leads to DNA-damage and apoptosis [2]. However, DNA-damage can also activate survival programs to manage the genotoxic stress, including NF-κB and MAPK [3]. Melphalan induced phosphorylation of TAK1, p38, JNK, ERK and the NF-κB transcription factor p65, and increased total p65-levels, indicative of MAPK and NF-κB activation. NG25 blocked melphalan-induced TAK1, p38, JNK and p65-phosphorylation, and blocked the increase in p65 levels, but induced further ERK-phosphorylation (Figure 2A). In addition, we observed a decrease in total TAK1 protein levels in melphalan+NG25 treated cells. In sum, NG25 blocks melphalan-induced p38, JNK and NF-κB signaling.

The serine-protein kinase ATM (ATM) is recruited to double-stranded DNA-breaks, and coordinates cell cycle arrest, survival pathways as well as initiation of intrinsic apoptosis [3]. Melphalan induced ATM-phosphorylation in INA-6 cells (Figure 2B, Supplementary Figure 6). We did not observe activation of mitochondrial apoptosis under these circumstances, as tested by BCL2 associated agonist of cell death (BAD) dephosphorylation or BH3 interacting domain death agonist (BID) and BCL2-like protein 11 (BIM) cleavage (Supplementary Figures 2 and 7). Thus, melphalan trigger ATM, and this might be responsible for TAK1-activation.

### NG25 reduce transcription of oncogenic transcription programs

**Figure 3:**
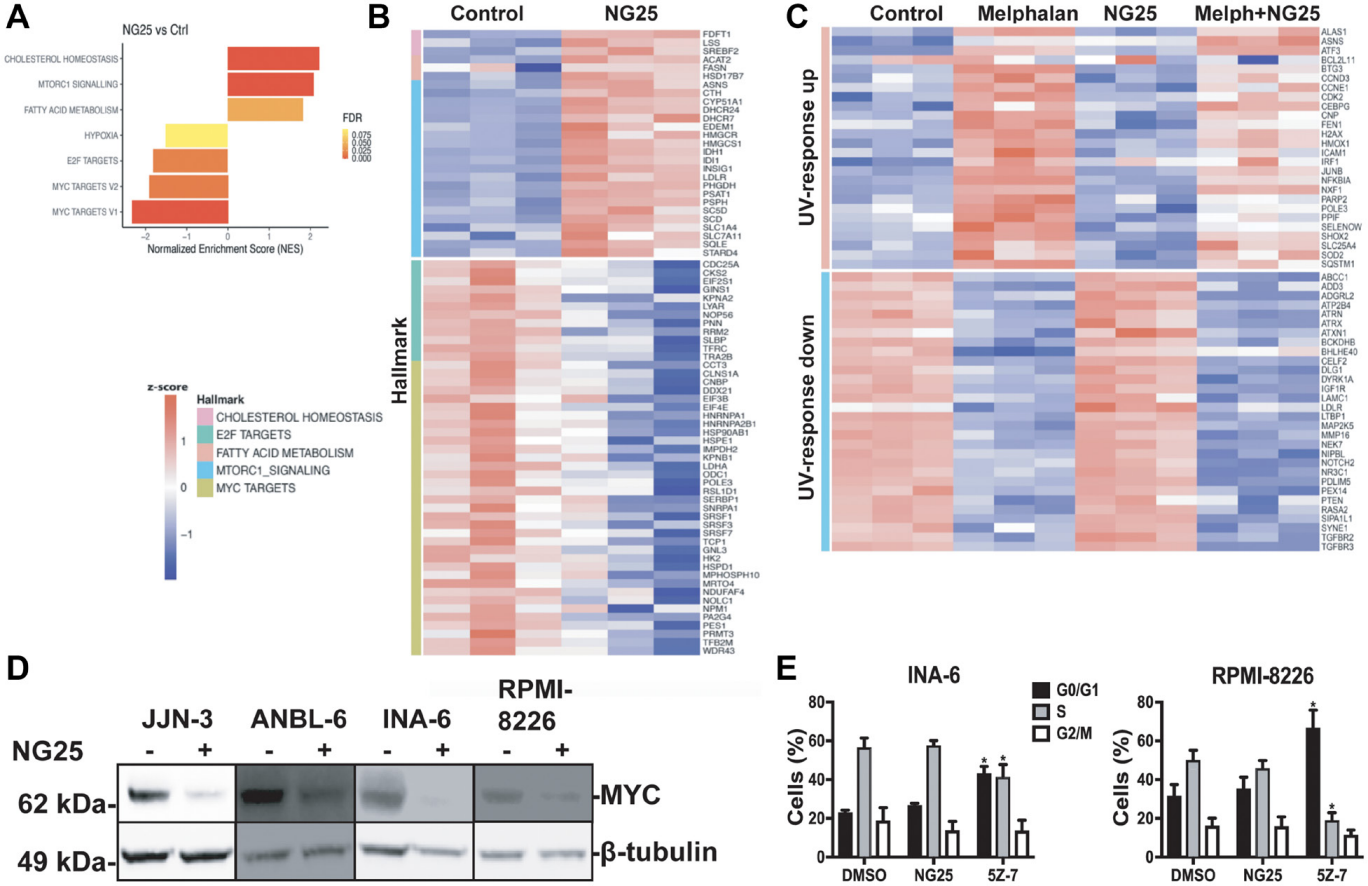
NG25 reduce transcription of oncogenic survival programs. (**A**–**C**) MM cells from the INA-6 cell line were treated with 2 μM NG25 or 10 μM melphalan or both for 6 hours. Then RNA was isolated, and the transcriptome was analyzed by RNA-sequencing. (A) Significantly changed pathways (FDR *q*-value < 0.02, NES > 1.3) in NG25-treated cells (*n* = 3) as compared to control (*n* = 3), analyzed by GSEA on hallmark gene sets. (B) Heatmap showing gene expression levels (z-score of log2 TPM) of genes included in the gene sets in (A). (C) Heatmap showing gene expression levels (z-score log2 TPM) of genes included in the UV-response gene sets, in cells treated with melphalan (*n* = 3) and/or NG25 (*n* = 3). (**D**) MM cell lines treated with 2 μM NG25 for 4 hours. Cell lysates were then analyzed for protein levels by immunoblotting. β-Tubulin is loading control. One representative of three independent experiments is shown, and full membranes from all experiments is given in Supplementary Figure 8. (**E**) INA-6 and RPMI-8226 MM cell lines were treated with 2 μM NG25 or 5Z-7 for 18 hours before fixation, PI-staining and flow cytometry analysis of cell cycle stage. The graph shows mean and SD from three independent experiments. Asterisks indicate significant differences (two-way ANOVA, Tukey’s multiple comparison test, *P* < 0,05) compared with DMSO. Histograms from one representative experiment is given in Supplementary Figure 3B.

MAP kinases and NF-κB regulate transcription of a multitude of genes. To determine which genes are affected by NG25 and melphalan, we performed an RNA-seq on INA-6 cells treated with NG25, melphalan or a combination of the two. GSEA focusing on the hallmark pathways showed that melphalan-treatment upregulated genes involved in UV-response, DNA-repair, p53-pathway and TNF-signaling via NF-κB (Supplementary Figure 3A). NG25 induced expression of genes involved in cholesterol homeostasis and mTORC1-signaling and down-regulated MYC- and E2F-targets (Figure 3A and 3B). MYC is a central driver of MM pathology, and regulate survival as well as cell cycle control through E2F [11, 12]. NG25 reversed the melphalan-induced UV-response (Figure 3C).

Consistent with the observation that NG25 decreased MYC-targets, NG25 also decreased MYC protein levels in MM cell lines, although not consistently in INA-6 (Figure 3D and Supplementary Figure 8). Of note, also melphalan reduced MYC protein levels (Supplementary Figure 8). We also performed a FACS-experiment to investigate whether the decreased MYC-activity had consequences for cell cycle progression in INA-6 and RPMI-8226 cells. NG25 did not affect cell cycle progression at the tested dose, whereas 5Z-7 treatment arrested cells in the G0/G1 phase (Figure 3E and Supplementary Figure 3B).

In sum, melphalan treatment induce pathways involved the DNA-damage response, and NG25 at least partly reverse these changes. In addition, NG25 down-regulates MYC which can render cancer cells less resilient to genotoxic stress.

### TAK1-inhibitors reduce numbers and viability of osteoclasts

**Figure 4:**
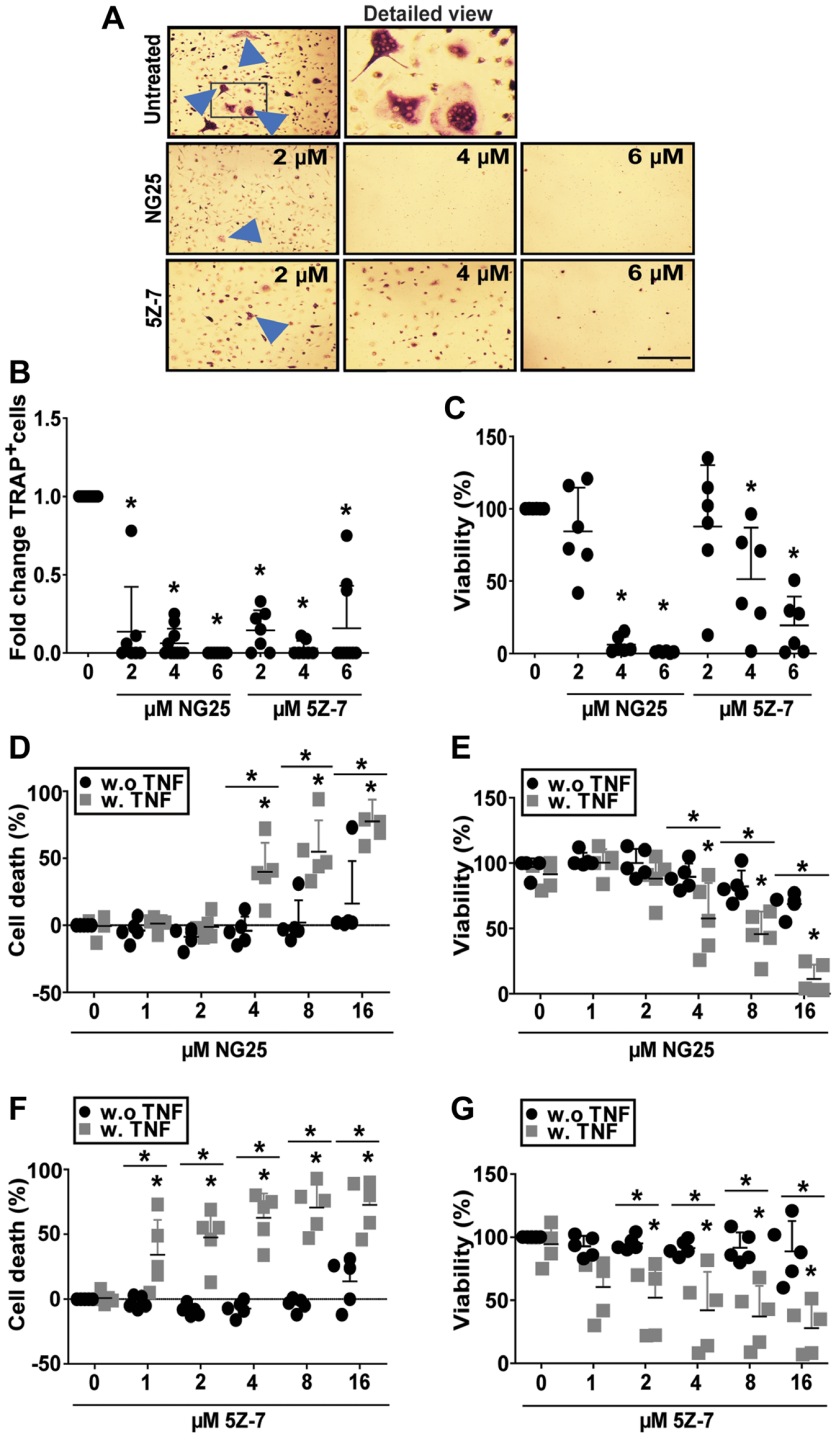
NG25 and 5Z-7 reduce viability and number of differentiating osteoclasts and trigger TNF-dependent cell death in pre-osteoclasts. (**A**–**C**) Human CD14^+^-monocytes were differentiated to OC, and treated with NG25 or 5Z-7 at indicated concentrations throughout differentiation, typically 10-15 days. (A) Representative phase-contrast images of TRAP-stained human osteoclasts, with arrowheads indicating multinuclear TRAP^+^ cells. Frame in first column indicates cutout for detailed view. Bar is 150 μM. (B) Fold change of TRAP^+^ multinuclear cells after NG25 or 5Z-7 treatment at indicated concentrations. 10 donors, mean, and SD are shown. (C) Viability at the time of TRAP^+^ quantification measured with the CellTiter-Glo assay. 6 donors, mean and SD are shown. (**D**–**G**) Pre-osteoclasts were treated with indicated concentrations of NG25 (D, E) or 5Z-7 (F, G) with or without 25 ng/ml TNF for 18 h and analyzed for cell death. Cell death was measured by LDH-release (D, F). Cell viability was measures with the CellTiter-Glo assay (E, G). 5 donors, mean and SD are shown. Asterisk denotes statistical significance between controls or indicated groups (B–G) (*P* < 0.05, two-way ANOVA).

Osteoclasts (OC) are specialized macrophages that degrade bone, and MM patients display elevated OC activity, leading to myeloma bone disease. We recently showed that inhibitors of the cellular inhibitor of apoptosis protein cIAP1/2, which acts upstream of TAK1 in several inflammatory pathways, reduced numbers and viability of human OC [13]. TAK1 was necessary for osteoclastogenesis in mouse [14]. On the basis of this we reasoned that TAK1-inhibitors also could block human osteoclastogenesis and have a double beneficial effect for MM patients. We found that treatment with NG25 and 5Z-7 during OC differentiation reduced both numbers and viability of human OC (Figure 4A–4C). We also tested whether TAK1-inhibitors induced a more rapid cytotoxicity on differentiated pre-OC. Here, TAK1-inhibitors were only cytotoxic in combination of a TAK1-activating agent such as TNF (Figure 4D–4G). In sum, TAK1-inhibitors can reduce osteoclast numbers through blocking the formation of new OC.

### NG25 reduce viability of myeloma patient samples alone and in combination with melphalan

**Figure 5:**
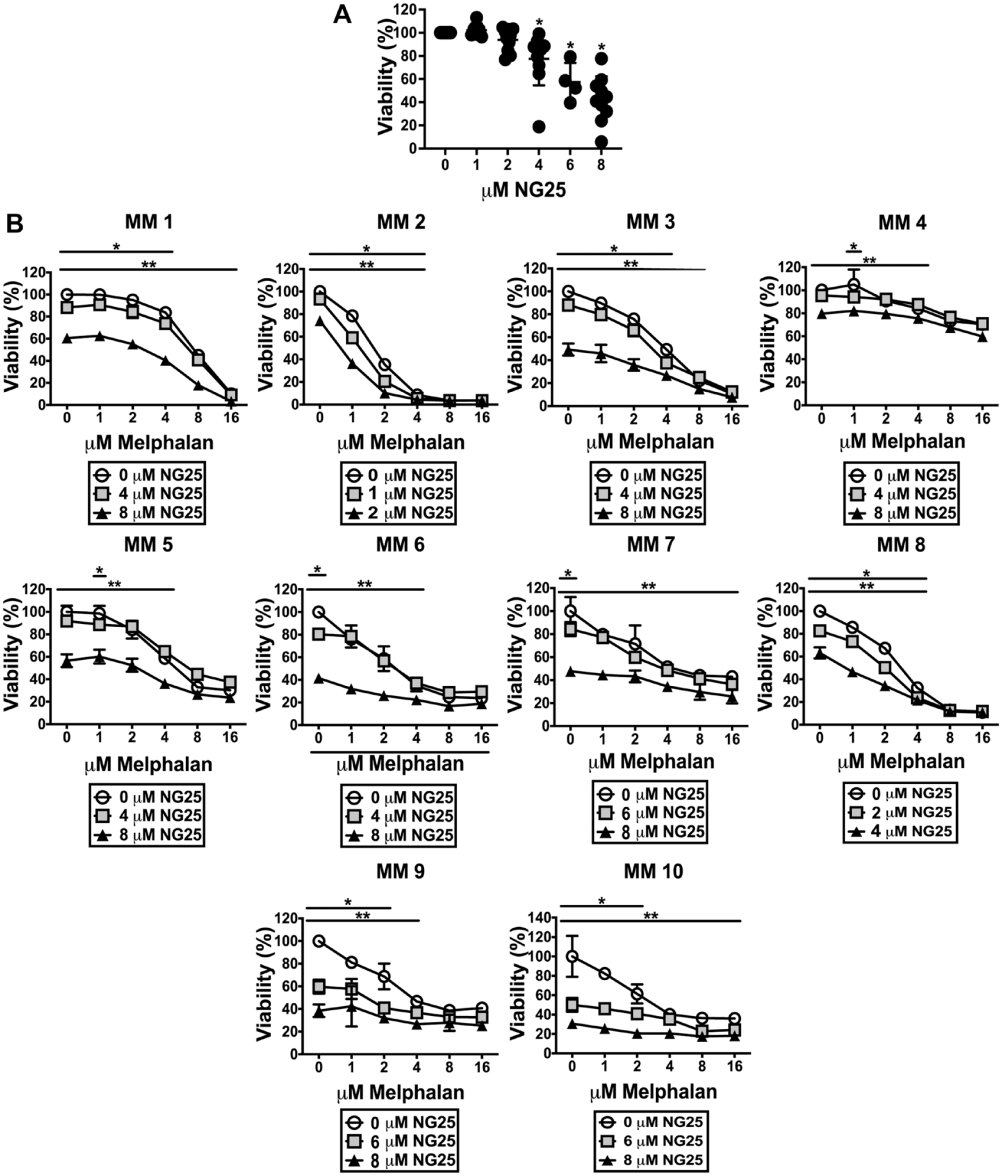
NG25 reduce viability of CD138^+^ cells from patients, alone and in combination with melphalan. (**A**) CD138^+^ cells from 10 individual donors were treated with NG25 before cell viability was measured. Individual donors, mean and SD are shown. Asterisks indicate statistically significant differences (Kruskal-Wallis test, Dunn's multiple comparison test, *P* < 0,05). (**B**) CD138^+^ cells from 10 individual donors were treated with melphalan, NG25 or both in technical triplicates. Mean and SD of triplicates from each donor are shown. Single asterisk denotes statistical significance as compared to the control not treated with NG25 for the lowest dose of NG25, double asterisk denotes statistical significance as compared to the untreated control for the highest dose of NG25. Asterisks indicate statistically significant differences (two-way ANOVA, Tukey’s multiple comparison test, *P* < 0,05). All treatments were for 18 hours, and viability was measured by CellTiter-Glo.

To investigate whether the observed cytotoxic effects of NG25 and melphalan also was evident in patient samples, we took advantage of our access to blood banked CD138^+^ plasma cells from myeloma patients. NG25 was cytotoxic to MM patient cells (Figure 5A and Supplementary Table 5). It also reduced the IC50 of melphalan, but the combined cytotoxicity was additive rather than synergistic (Figure 5B, Supplementary Table 6).

## DISCUSSION

In this study, we present findings showing that TAK1-inhibitors are cytotoxic to multiple myeloma cells and OC. Further, TAK1-inhibitors potentiated the cytotoxicity of melphalan and blocked melphalan-induced NF-κB and MAPK activation. NG25 reversed the melphalan-induced UV-response. On the basis of this we suggest that combining TAK1-inhibitors with alkylating agents such as melphalan and cyclophosphamide can be a beneficial combination for MM treatment.

NG25 reduced activity of oncogenic drivers such as MYC and E2F, but up-regulated genes involved in mTORC1-signaling and cholesterol homeostasis. The latter is consistent with previous findings that TAK1 knockout induce increased mTORC1-activity and hepatosteatosis in mice [15]. NG25 also neutralized the melphalan-induced transcriptional UV-response. In sum, TAK1-inhibitors can target MM cells in several manners: i) directly shifting constitutively- or melphalan-activated TAK1 from NF-κB/MAPK activation to cell death, ii) reducing activity of survival factors such as MYC and E2F, and iii) neutralizing the melphalan-induced UV-response.

Bone disease is a severe complication of MM. It causes pain for the patients and promotes tumor growth. The TAK1/RIPK1 pathway is central in regulation of inflammatory activation and death of macrophages, and we recently showed that Smac-mimetics, which work directly upstream of TAK1, blocked osteoclastogenesis and shifted OC to cell death [13]. Previous work showed that TAK1 is involved in mouse osteoclastogenesis, and that 5Z-7 was cytotoxic to mouse OC [9, 10, 14]. Here we show that this is conserved in human OC. Both 5Z-7 and NG25 blocked osteoclastogenesis and was cytotoxic to differentiating OC at higher doses. TAK1-inhibitors thus have an additional antitumorigenic effect through blocking osteoclastogenesis and potentially limiting MBD.

In sum, TAK1-inhibitors reduce MM cell viability and induce apoptosis through several mechanisms. They potently induce cell death in combination with melphalan and reduce OC numbers and viability. TAK1 is an interesting candidate for further clinical testing as a drug target in MM.

## MATERIALS AND METHODS

### Cell culture

The myeloma cell lines used in this study were ANBL-6 (a kind gift from Dr. Diane Jelinek, Mayo Clinic, Rochester, MN, USA), INA-6 (a kind gift from Dr. Martin Gramatzki, Erlangen, Germany), JJN-3 (a kind gift from Dr. Jennifer Ball, University of Birmingham, UK), and RPMI-8226 (from ATCC, Rockville, MD, USA). ANBL-6 and INA-6 cells were grown in 10% heat-inactivated fetal calf serum (FCS) in RPMI-1640 (hereafter described as RPMI, Sigma-Aldrich, St. Louis, MO, USA, R8758) supplemented with recombinant human interleukin-6 (IL-6, 1 ng/ml) (Gibco, Life Technologies/Thermo Fisher Scientific, Waltham, MA, USA). JJN-3 cells were maintained in 10% heat-inactivated FCS in RPMI-1640. RPMI-8226 cells were maintained in 20% heat-inactivated FCS in RPMI-1640. All cells were cultured at 37°C in a humidified atmosphere containing 5% CO_2_.

To obtain primary myeloma cells, CD138^+^ cells were isolated from bone marrow specimens obtained through the Norwegian Myeloma Biobank (Biobank1) using RoboSep automated cell separator and Human CD138 Positive Selection Kit (StemCell Technologies, Grenoble, France). Informed consent was obtained from participating patients, and the regional ethics committee approved the study (REK Midt 2011/2029). Experiments with primary myeloma cells, were performed in 2% heat-inactivated human serum (Blood Bank, St Olav’s University Hospital, Trondheim, Norway) supplemented with recombinant human IL-6 (1 ng/ml).

To generate primary human osteoclasts, peripheral blood mononuclear cells were isolated from healthy donors by density centrifugation. Buffy coats were provided by the Blood Bank (St. Olavs Hospital, Trondheim) with approval by the Regional Committee for Medical and Health Research Ethics (REK Midt 2011/2029). Osteoclasts (OC) and pre-osteoclasts (pre-OC) was generated from PBMCs as described previously [13]. In brief, monocytes were isolated using CD14^+^ magnetic bead separation according to the manufacturer’s instructions (Miltenyi Biotec, Bergisch Gladbach, Germany, 130-050-201). CD14^+^ monocytes were plated in α minimum essential medium (αMEM, Thermo Fisher Scientific, 41061-029) supplemented with 10% heat-inactivated human serum according to blood type, 30 ng/ml CSF-1 (216-MC), 10 ng/ml RANKL (390-TN-010), and 1 ng/ml TGF-β (240-B-002) (R&D systems, Minneapolis, MN, USA) [13, 16]. The cells were differentiated for 6–7 days to obtain osteoclast precursors (pre-OC), and until visible multinuclear cells for osteoclasts, typically 10–15 days.

The following compounds were used in cell culture: 5Z-7 (Sigma-Aldrich, O9890), doxorubicine (Sigma-Aldrich, D1515), etoposide (Sigma-Aldrich, E1383), NG25 (MedChemExpress, Monmouth Junction, NJ, USA, HY-15434), melphalan (Sigma-Aldrich, St. Louis, MO, USA, M2011), recombinant human TNF (R&D Systems, 410-MT-025).

### Assessment of osteoclast differentiation

The number of mature osteoclasts was assessed by scoring the number of multinucleated cells (≥ 3 nuclei) that were positive for tartrate resistant acid phosphatase (TRAP). When multinucleated cells were visible by light microscopy, cells were fixed and stained for TRAP using the Leukocyte Acid Phosphatase Kit (Sigma-Aldrich, 387A-1KT) according to the manufacturer’s instructions.

### Cell lysis and western blotting

Cells were sedimented and washed in PBS before lysis 15 minutes on ice (50 mM tris-HCl, TritonX-100 (1%), 150 mM NaCl, 5 mM EDTA, protease inhibitor cocktail (Roche, Basel, Switzerland, 1187358001), 1 mM Na_3_VO_4_ and 50 mM NaF. The samples were separated on NuPAGE Bis-Tris gels with MOPS or MES running buffer (Invitrogen, Thermo Fisher Scientific). Proteins were transferred from the gel onto a 0.2 μm nitrocellulose membrane using the iBlot gel transfer system (Life Technologies, Thermo Fisher Scientific). The membrane was blocked with 5% BSA in tris-buffered saline with 0.1% Tween 20 (TBS-T) and incubated with primary antibodies. Blots were washed with TBS-T before incubation with horse-radish peroxidase- or fluorophore-conjugated secondary antibodies (Dako, Agilent, Santa Clara, CA, USA and LiCor Biosciences, Lincoln, NE, USA). Membranes were analyzed on a LiCor Fc or xCT (LiCor Biosciences). For luminescence, a SuperSignal West Femto luminescence substrate was used (Thermo Fisher Scientific, 34096).

The following antigens were analyzed (antibodies in parenthesis): ATM phospho-Ser1981 (Cell Signaling Technologies (CST), Danvers, MA, USA, 5883), Bad phospho-Ser112 (CST, 5284), Bad (CST, 9239), Bak (CST, 12105), Bax (CST, 5023), BID (CST, 2002), Bim (CST, 2933), β-tubulin (Abcam, Cambridge, UK, Ab6046), Caspase 3 (CST, 9662), Caspase 8 (Enzo, ALX-804-242-C100), Caspase 8 (CST 9748), Caspase 9 (CST, 9502), MYC (CST, 5605), ERK1/2 phospho-Thr202/Tyr204 (CST, 4370), ERK1/2 (CST, 9107), GAPDH (CST, 2118), JNK phospho-Thr183/185 (CST, 4668), JNK, PARP (CST, 9532), p38 phospho-Thr180/Tyr182 (CST, 9215), p38 (CST, 9212), p65 phospho-Ser536 (CST, 3033), p65 (CST, 6956), TAK1 phospho-Thr184/187 (4508), TAK1 (CST, 4505).

### Cytotoxicity and viability assays

For viability assessment of MM cells, CellTiter-Glo^®^ 2.0 Cell Viability Assay (Promega Madison, WI, USA G358C) was used to measure the levels of ATP in the wells according to the manufacturer›s instructions. 5000 cells of MM cell lines and 10 000 cells of CD138^+^ primary MM cells were seeded in 96-well plates and stimulated as indicated and then incubated for 18 hours at 37°C in humidified atmosphere containing 5% CO_2_. Luminescence was measured with a Victor 1420 multilabel counter (Perkin Elmer Inc., Waltham, MA, USA). All conditions were done in triplicates, and all experiments were performed at least three times.

For cell death assessment of pre-OC, cells were stimulated in Opti-MEM serum free medium (Thermo Fisher Scientific, 11058-021), and cell death was calculated by measuring lactate dehydrogenase-release (LDH) in medium using a colorimetric assay according to the manufacturer’s instructions (TaKaRa Bio, Saint-Germain-en-Laye, France, MK401). Cell viability of OC and pre-OC was measured using the colorimetric Cell titer 96^®^ AQueous One Solution Cell Proliferation Assay (Promega, G358C) or the luminescent CellTiter-Glo^®^ 2.0 Cell Viability Assay.

### Cell cycle analysis

INA-6 and RPMI-8226 cells were treated with TAK1-inhibitors or vehicle for 18 hours. Cells were then washed in PBS and fixated in ice cold methanol. Methanol was removed, cells were washed in PBS, and incubated in RNAse for 30 minutes at 37°C to remove RNA, before propidium iodide was added and cells incubated for 30 minutes at 37°C. Cells were then filtered with a 40 μM filter. Data were acquired using a LSRII flow cytometer (BD Biosciences, San Jose, CA, USA) and analyzed in FlowJo (Tree Star, Inc., OR, USA) using the Watson pragmatic model.

### Survival and progression-free survival analysis of CoMMpass gene expression data

RNA sequencing data of CD138^+^ cells from 773 patients were available from the Multiple Myeloma Research Foundation CoMMpass Researcher Portal (IA15-release). Patient samples were divided into high and low *MAP3K7* expression based on the upper 20th percentile (*n* = 154 patients) and lower 80th percentile (*n* = 619 patients), and the CoMMpass researcher portal analysis tool was used to generate Kaplan-Meyer plots and hazard-ratio of progression-free survival and survival.

### Transcriptome sequencing

INA-6 cells were treated with 2 μM NG25 or 10 μM melphalan or both for 6 hours. Total RNA was then extracted using RNeasy Mini kits including DNAse digestion with Qiacube (QIAGEN, Hilden, Germany, 74104). RNA quantity, quality/integrity and purity were evaluated using Qubit (Thermo Fisher Scientific, MA, USA), Bioanalyzer (Agilent, CA, USA) and NanoDrop (Thermo Fisher Scientific, MA, USA).

RNA sequencing libraries were generated for 12 RNA samples, using SENSE mRNA-Seq library prep kit V2, according to manufacturer’s instructions (Lexogen GmbH, Vienna, Austria). In brief, 800 ng of total RNA was prepared and incubated with magnetic beads coated with oligo-dT, then all other RNAs except mRNA were removed by washing. Library preparation was then initiated by random hybridization of starter/stopper heterodimers to the poly(A) RNA still bound to the magnetic beads. These starter/stopper heterodimers contain Illumina-compatible linker sequences. A single-tube reverse transcription and ligation reaction extends the starter to the next hybridized heterodimer, where the newly synthesized cDNA insert was ligated to the stopper. Second-strand synthesis was performed to release the library from the beads. The resulting double-stranded library was purified and amplified (12 PCR cycles) after adding the adaptors and indexes. Finally, libraries were quantitated by qPCR using KAPA Library Quantification Kit (Kapa Biosystems, Inc., MA, USA) and validated using Agilent High Sensitivity DNA Kit on a Bioanalyzer (Agilent Technologies, CA, USA). The size range of the DNA fragments were measured to be in the range of 200-450 bp with average library size 240 bp.

Prior to sequencing, the libraries were quantitated by quantitative polymerase chain reaction using the KAPA Library Quantification Kit–Illumina/ABI Prism® (Kapa Biosystems, Wilmington, MA, USA) and validated using the Agilent High Sensitivity DNA Kit on a bioanalyzer. Libraries were normalized to 2.7 pM subjected to clustering. Single read sequencing was performed for 75 cycles on a NextSeq500 HO flowcell (Illumina, San Diego, CA, USA), according to the manufacturer’s instructions. Base calling was done on the NextSeq500 instrument by RTA 2.4.6. FASTQ files were generated using bcl2fastq2 Conversion Software v2.20.0.422 (Illumina, Inc.).

FASTQ files were filtered and trimmed (fastp v0.20.0) and transcript counts were generated using quasi alignment (Salmon v1.3.0) to the transcriptome reference sequence (Ensembl, GRCh38 release100). Transcript sequences were imported into the R statistical software and aggregated to gene counts using the tximport (v1.14.0) bioconductor package. Data are available in the GEO Repository under accession number GSE178292.

For differential expression analysis, a DESeq2 unpaired analysis were performed in R studio to find differentially expressed genes. Gene Set Enrichment Analysis (GSEA; Broad Institute) was used to identify enriched pathways.

### Statistical analysis

Statistical analysis was performed using the GraphPad Prism 6 software (GraphPad Software Inc., La Jolla, CA, USA). The tests used were one-way ANOVA with Tukey’s multiple comparison test, Kruskal-Wallis test with Dunn’s multiple comparison test, or two-way ANOVA with Tukey’s multiple comparison test. For dose-response curves, significant differences in response were calculated as follows: data were normalized within groups and IC50 was calculated by linear regression (non-linear regression, log(inhibitor) vs response, variable slope). Level of statistical significance was set at 0.05 (5%) for all experiments.

## SUPPLEMENTARY MATERIALS



## Abbreviations

5Z-7: 5Z-7-oxozeaenol
ATM: serine-protein kinase ATM
BAD: BCL2 associated agonist of cell death
BID: BH3 interacting domain death agonist
BIM: BCL2-like protein 11
E2F: transcription factor E2F
ERK: extracellular-signal-regulated-kinase
CD138: cluster of differentiation 138
cIAP: cellular inhibitor of apoptosis protein
FACS: fluorescence associated cell sorting
JNK: c-Jun N-terminal kinase
MAPK: mitogen activated protein kinase
MAP3K7: mitogen activated protein protein protein kinase 7
MBD: myeloma bone disease
MM: multiple myeloma
MRD: minimal residual disease
mTORC1: mammalian target of rapamycin complex 1
NF-κB: nuclear factor kappa-light-chain-enhancer of activated B cells
OC: osteoclast
PARP: poly [ADP-ribose] polymerase
pre-OC: pre-osteoclast
RIPK1: receptor-interacting protein kinase 1
TAK1: transforming growth-factor-β activated kinase 1
TNF: tumor necrosis factor
