# Supplementary material for: TAK1-inhibitors are cytotoxic for multiple myeloma cells alone and in combination with melphalan
Source: Oncotarget. 2021 Oct 12;12(21):2158–68. doi: 10.18632/oncotarget.28073 (PMC8522844; doi:10.18632/oncotarget.28073)
Supplement: Supplementary file 1 [file oncotarget-12-2158-s001.pdf]

TAK1-inhibitors are cytotoxic for multiple myeloma cells alone and in combination with melphalan

SUPPLEMENTARY MATERIALS

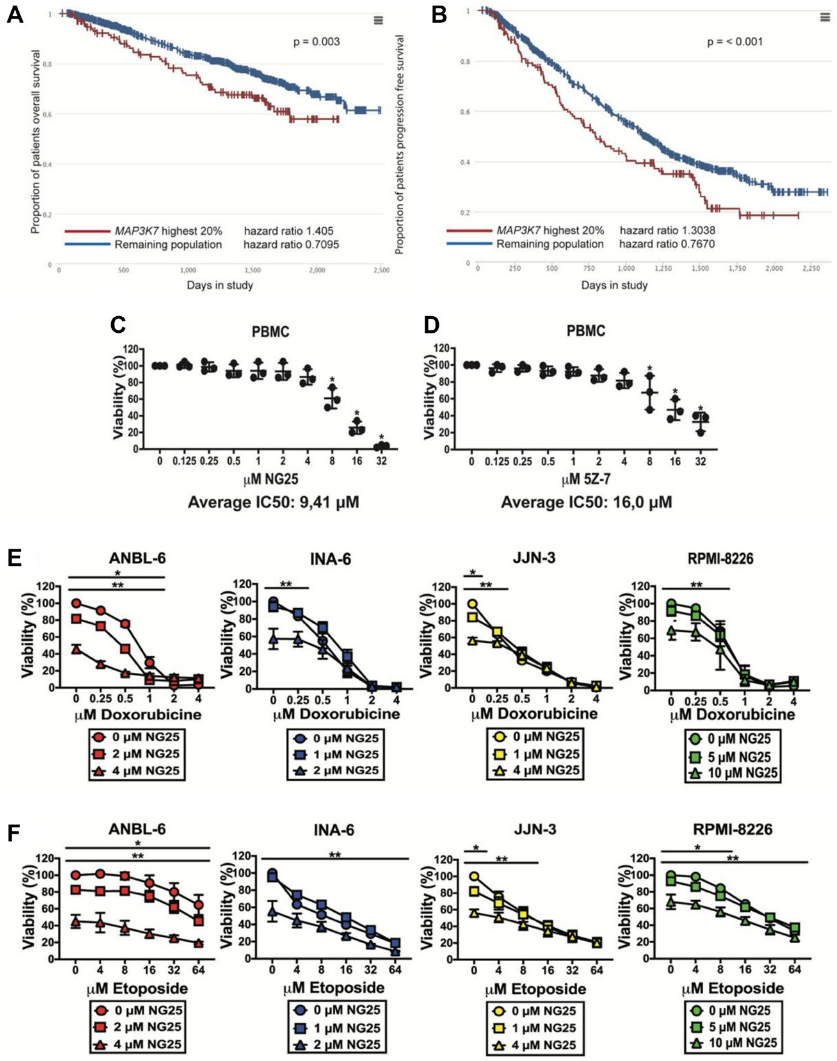

Supplementary Figure 1: NG25 is cytotoxic in PBMCs and in combination with DNA-damaging drugs in MM cell lines. (**A**) Survival and (**B**) progression-free survival curves generated from the CoMMpass data (IA15 Release) by comparing the upper 20th percentile MAP3K7 expressing patients with the lower 80th percentile. Log-rank P is 0.003 (A) and < 0.001 (B). (**C**) Three PBMC donors treated with NG25 in triplicates at indicated concentrations for 18 hours before measuring the viability with CellTiter-Glo. Asterisks indicate statistically significant differences (One-way ANOVA, Tukey's multiple comparison test, $P < 0,05$) compared with untreated. (**D**) Three PBMC donors treated with 5Z-7 in triplicates at indicated concentrations for 18 hours before measuring the viability with CellTiter-Glo. Asterisks indicate statistically significant differences (One-way ANOVA, Tukey's multiple comparison test, $P < 0,05$) compared with untreated. (**E**) MM cell lines treated with NG25 in combination with doxorubicine. Asterisks indicate statistically significant differences (two-way ANOVA, Tukey's multiple comparison test, $P < 0,05$) compared with the control not treated with NG25. Single asterisk denotes statistical significance as compared to the control not treated with NG25 for the lowest dose of NG25, double asterisk denotes statistical significance as compared to the untreated control for the highest dose of NG25. (**F**) MM cell lines treated with NG25 in combination with etoposide. Asterisks indicate statistically significant differences (two-way ANOVA, Tukey's multiple comparison test, $P < 0,05$) compared with the control not treated with NG25. Single asterisk denotes statistical significance as compared to the control not treated with NG25 for the lowest dose of NG25, double asterisk denotes statistical significance as compared to the untreated control for the highest dose of NG25.

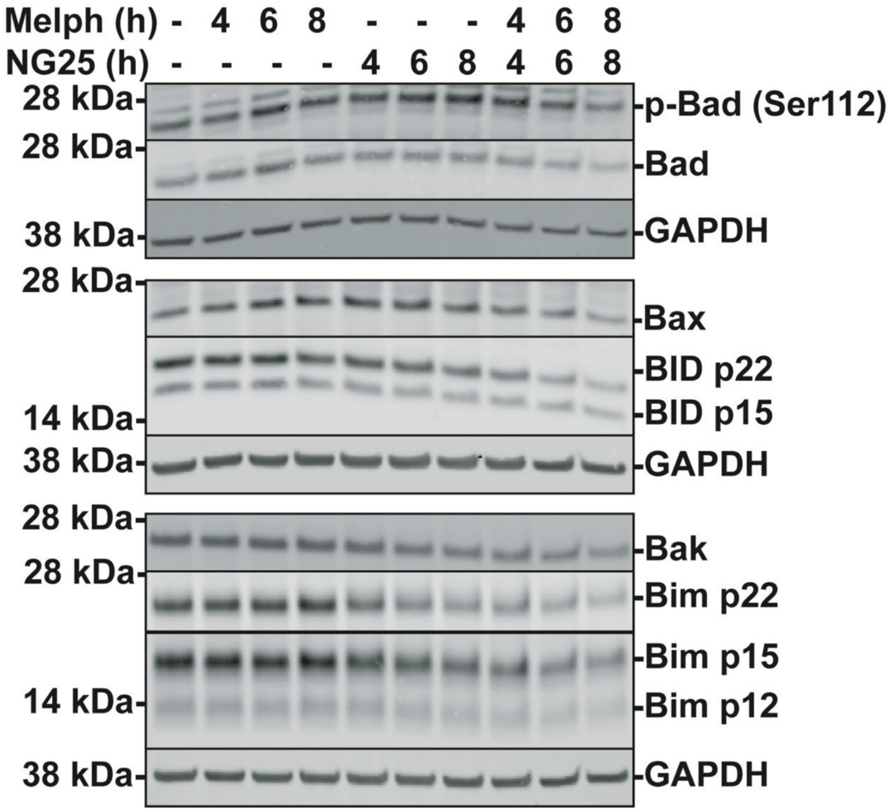

Supplementary Figure 2: Mitochondrial directed apoptosis is not activated during these experimental conditions. MM cells from the INA-6 cell line were treated with 2 μM NG25, 10 μM melphalan or both for the indicated time points (hours, h), and cell lysates were analyzed for protein levels by immunoblotting. GAPDH is loading control, shown for corresponding membranes. One representative of two independent experiments is shown. Full membranes for both experiments are given in Supplementary Figure 7.

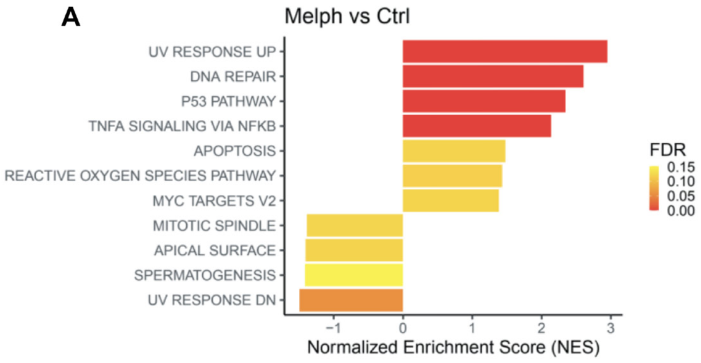

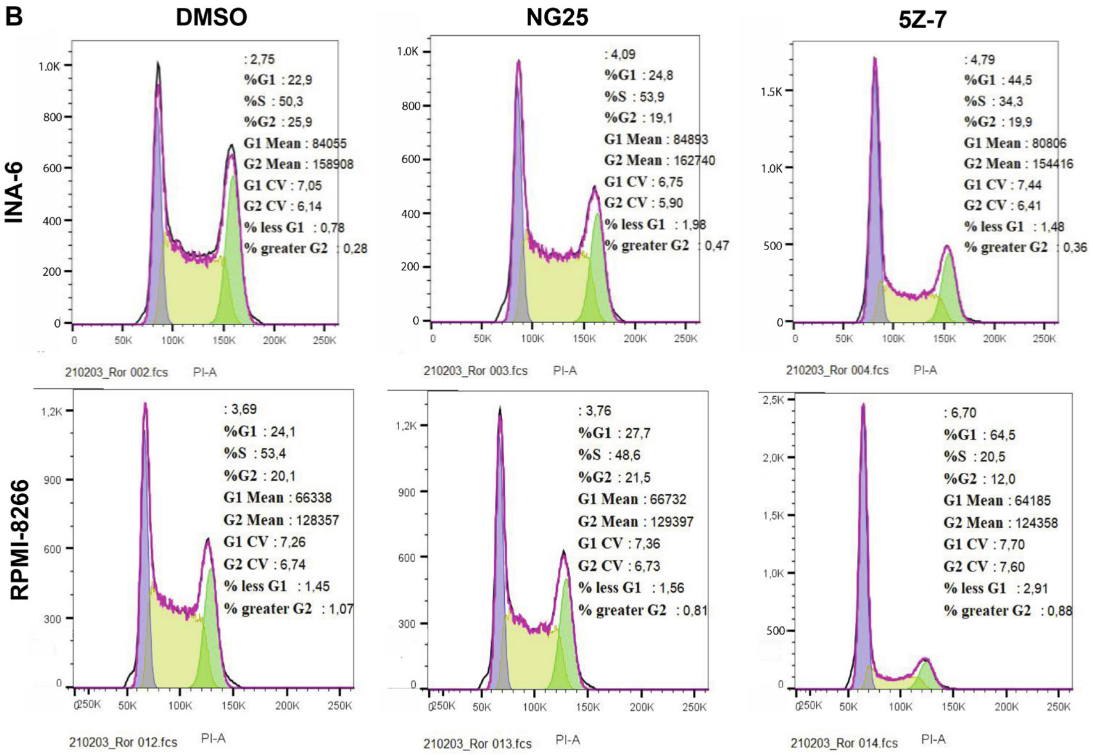

Supplementary Figure 3: Melphalan-induced gene expression patterns and flow-histograms from cell cycle analysis. (**A**) GO-enrichment plot of INA-6 cells treated with 10 µM melphalan as compared to untreated controls. (**B**) FlowJo printout of histograms and cell cycle stage population gating using the Watson pragmatic model. One representative experiment is shown.

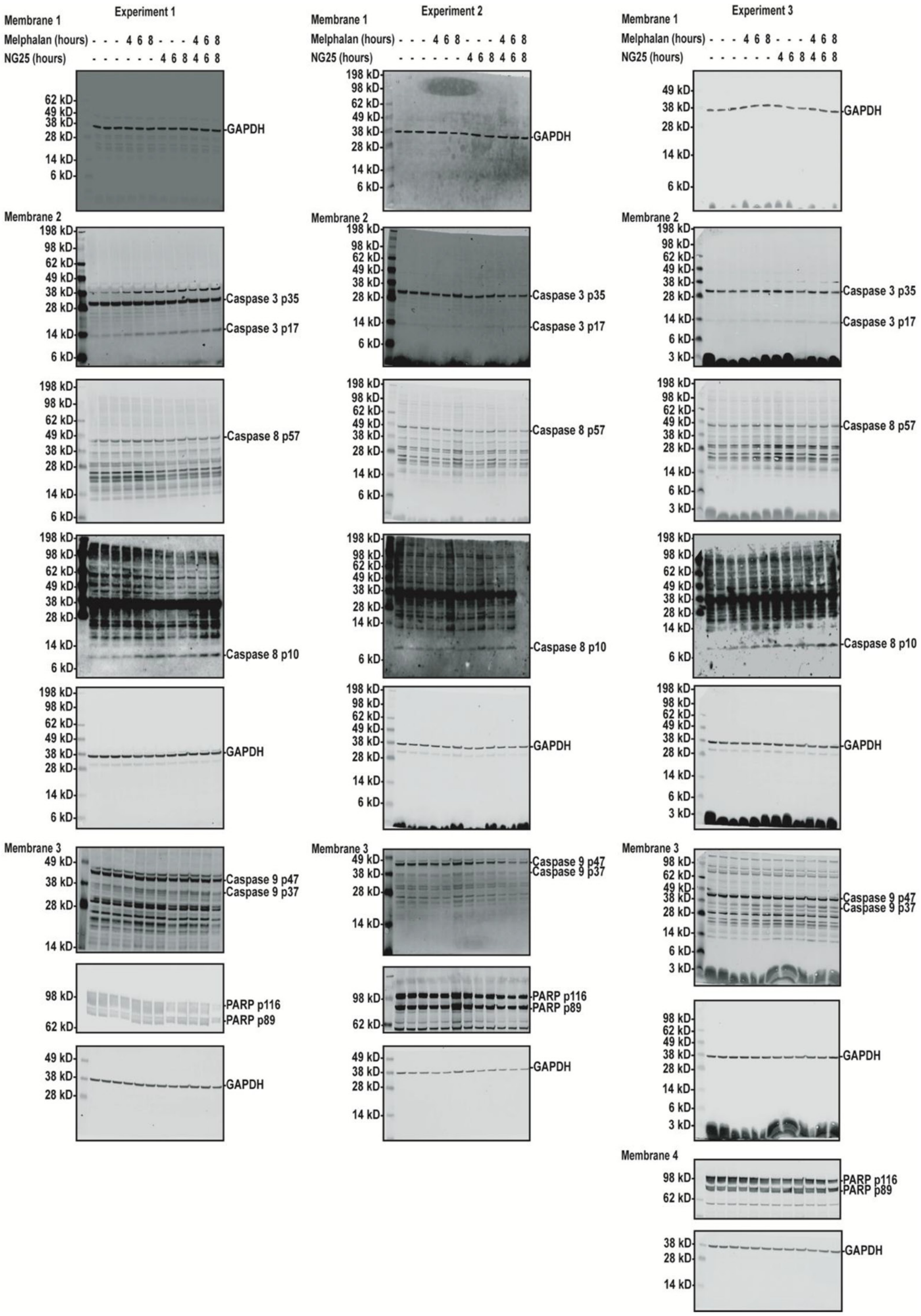

Supplementary Figure 4: Total experiments and full immunoblots for NG25 and melphalan effect on cell death signaling. INA-6 cells were treated with 2 μM NG25, 10 μM melphalan or both for the indicated time points (hours) and cell lysates were analyzed for caspase 3, caspase 8, caspase 9, and PARP protein levels by immunoblotting. GAPDH is loading control. Experiment 1 is same is in Figure 1E.

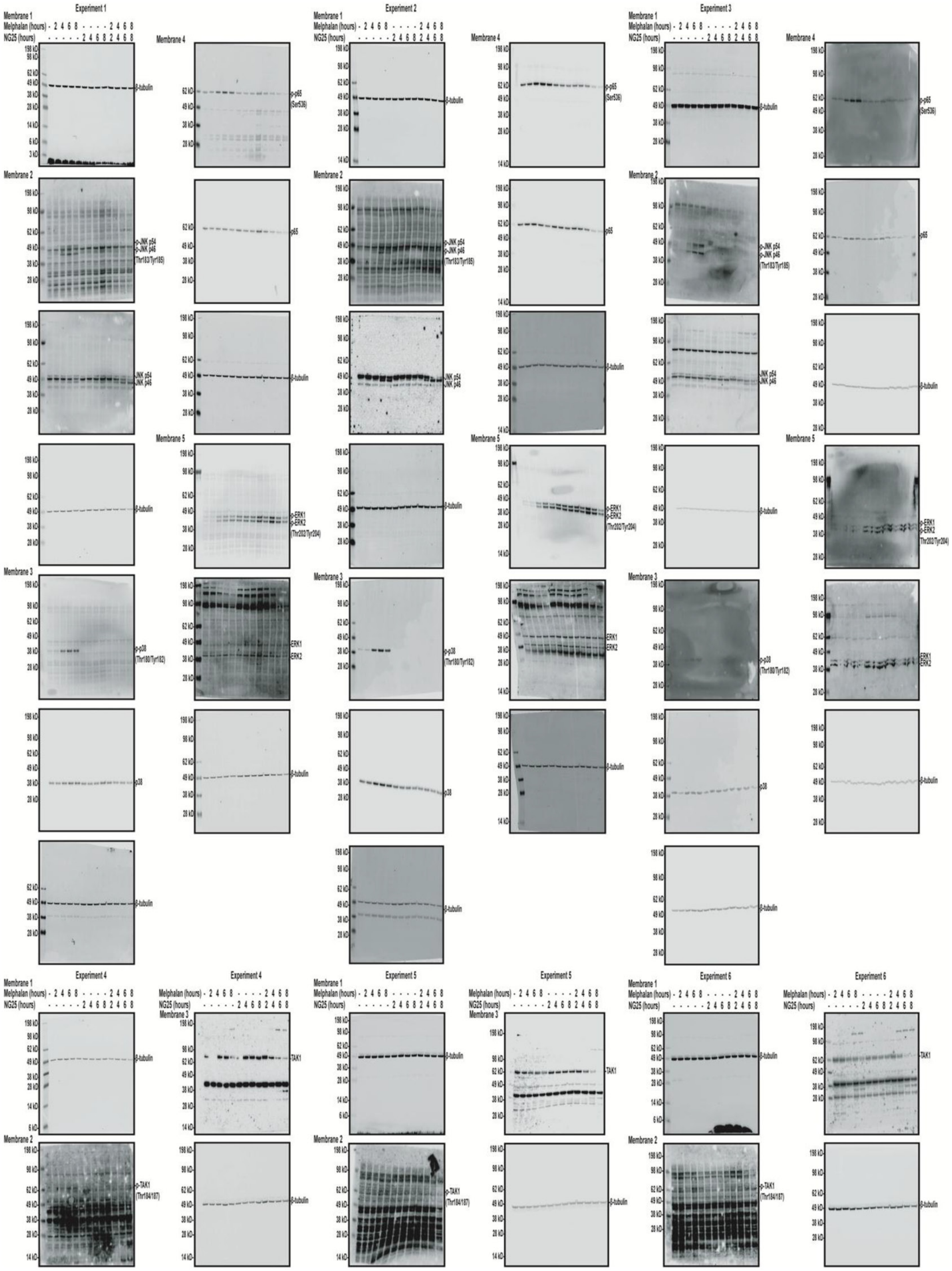

Supplementary Figure 5: Experiments and full immunoblots for NG25 and melphalan effect on MAPK and NF-κB. INA-6 cells were treated with 2 μM NG25 or 10 μM melphalan or both at the indicated time points, and cell lysates were analyzed for the indicated antigen levels by immunoblotting. β-Tubulin is loading control. Experiment 1 and 5 is same as in Figure 2A.

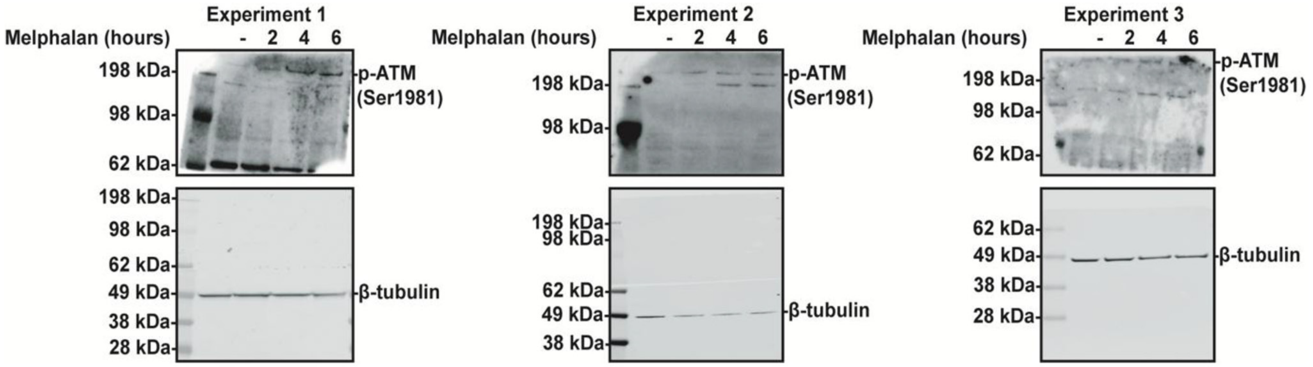

Supplementary Figure 6: Experiments and full immunoblots for NG25 and melphalan effect on ATM phosphorylation and activation. INA-6 cells line were treated with 10 μM melphalan at the indicated time points, and cell lysates were analyzed for phospho-Ser1981-ATM protein levels by immunoblotting. β-Tubulin is loading control. Experiment 1 is same as in Figure 2B.

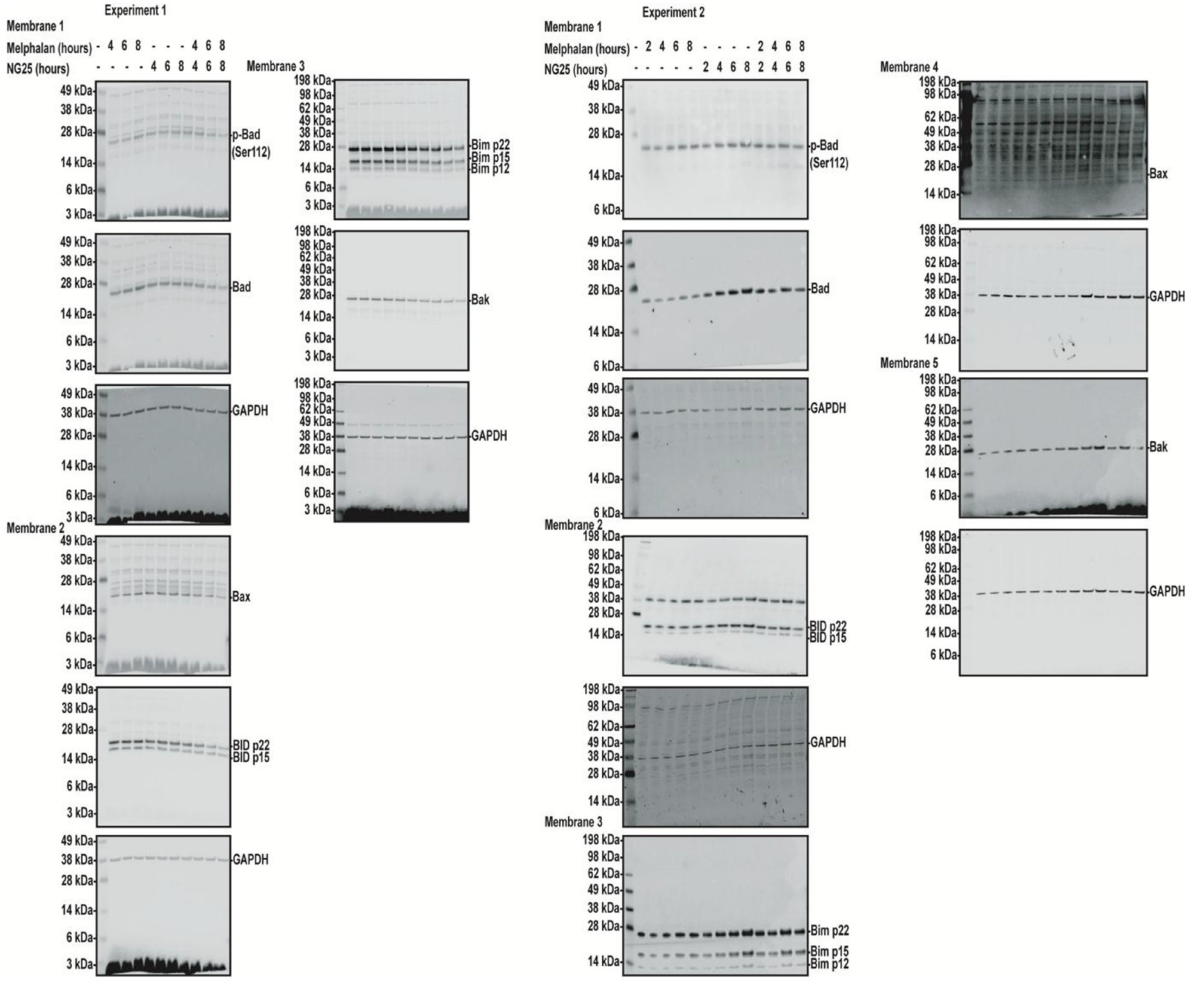

Supplementary Figure 7: Total experiments and full immunoblots for NG25 and melphalan effect on Bcl-2 family proteins. INA-6 cells were treated with 2 μM NG25 or 10 μM melphalan or both at the indicated time points, and cell lysates were analyzed protein levels of the indicated antigens by immunoblotting. GAPDH is loading control. Experiment 1 is same as in Supplementary Figure 2.

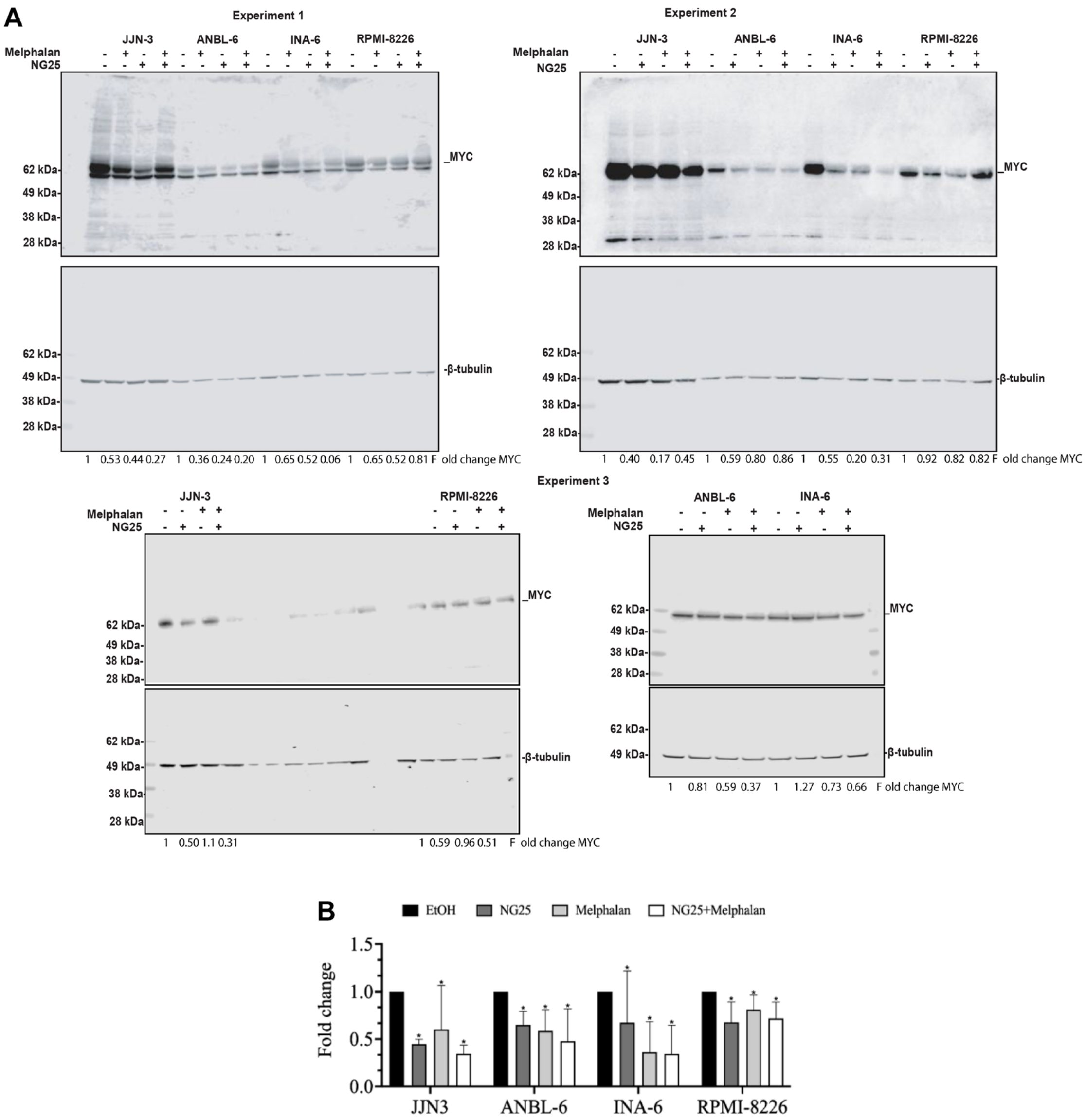

Supplementary Figure 8: MYC all membranes. (**A**) MM cells from the JJN-3, ANBL-6, INA-6 and RPMI-8226 cell lines were treated with 2 μM NG25 for 4 hours, and cell lysates were analyzed for MYC protein levels by immunoblotting. β-Tubulin is loading control. Relative MYC protein levels (adjusted for loading) are given as fold change from untreated control. Experiment 2 is same as is Figure 3D. (**B**) Average fold change of MYC protein levels; average and standard deviation is shown. Asterisk indicate statistical difference as compared to the untreated control ($P < 0.05$, one-way ANOVA, Tukey's Multiple Comparison test).

Supplementary Table 1: IC50 TAK1-inhibitors PBMC

	Donor 1	Donor 2	Donor 3	Average
IC50 (µM NG25)	11,1	9,11	8,02	9,41
IC50 (µM 5Z-7)	23,1	16,4	8,55	16,0

PBMCs were treated with TAK1-inhibitors for 18 hours, and cell viability was assessed by Cell Titer viability assay. Results are average of technical triplicates for each donor.

Supplementary Table 2: IC50 melphalan + TAK1-inhibitors in MM cell lines

Cell line	Treatment	IC50 (µM melphalan)
INA-6	0 µM NG25	4,94
	2 µM NG25	2,70
	4 µM NG25	0,66
ANBL-6	0 µM NG25	7,69
	2 µM NG25	3,45
	4 µM NG25	1,29
JJN-3	0 µM NG25	12,5
	1 µM NG25	9,59
	4 µM NG25	5,59
RPMI-8226	0 µM NG25	24,5
	5 µM NG25	14,4
	10 µM NG25	9,44
INA-6	0 µM 5Z-7	4,92
	1 µM 5Z-7	2,94
	2 µM 5Z-7	2,25
ANBL-6	0 µM 5Z-7	7,73
	1 µM 5Z-7	5,16
	2 µM 5Z-7	3,89
JJN-3	0 µM 5Z-7	15,5
	0,5 µM 5Z-7	12,9
	2 µM 5Z-7	6,44
RPMI-8226	0 µM 5Z-7	23,8
	2 µM 5Z-7	14,9
	8 µM 5Z-7	3,37

The indicated MM cell lines were treated with melphalan and the indicated doses of NG25 for 18 hours, before cell viability was assessed by Cell Titer viability assay. Results are average of three independent experiments.

Supplementary Table 3: IC50 NG25 + doxorubicine in MM cell lines

Cell line	µM NG25	IC50 (µM doxorubicine)
ANBL-6	0 µM NG25	0,73
	2 µM NG25	0,43
	4 µM NG25	0,02
INA-6	0 µM NG25	0,54
	1 µM NG25	0,73
	2 µM NG25	0,36
JJN-3	0 µM NG25	0,35
	1 µM NG25	0,41
	4 µM NG25	0,31
RPMI-8226	0 µM NG25	0,63
	5 µM NG25	0,59
	10 µM NG25	0,41

The indicated MM cell lines were treated with doxorubicine and the indicated doses of NG25 for 18 hours, before cell viability was assessed by Cell Titer viability assay. Results are average of three independent experiments.

Supplementary Table 4: IC50 NG25 + etoposide in MM cell lines

Cell line	µM NG25	IC50 (µM etoposide)
ANBL-6	0 µM NG25	97,9
	2 µM NG25	58,2
	4 µM NG25	2,24
INA-6	0 µM NG25	8,70
	1 µM NG25	14,2
	2 µM NG25	3,33
JJN-3	0 µM NG25	11,2
	1 µM NG25	10,2
	4 µM NG25	4,34
RPMI-8226	0 µM NG25	31,4
	5 µM NG25	31,6
	10 µM NG25	11,4

The indicated MM cell lines were treated with etoposide and the indicated doses of NG25 for 18 hours, before cell viability was assessed by Cell Titer viability assay. Results are average of three independent experiments.

Supplementary Table 5: IC50 NG25 in primary myeloma cells

Donor	IC50 (µM NG25)
MM1	9,37
MM2	2,74
MM3	8,01
MM4	13,8
MM5	8,57
MM6	6,92
MM7	7,68
MM8	4,96
MM9	6,79
MM10	5,92
**Average IC50**	**7,48**

$CD138^{+}$ cells from myeloma patients were treated with NG25 for 18 hours, and cell viability was determined by Cell Titer Viability assay. Results are average of technical triplicates for each donor.

Supplementary Table 6: IC50 for melphalan + NG25 in primary myeloma cells

Donor	Treatment	IC50 (µM melphalan)
MM 1	0,0 µM NG25	7,36
	2,0 µM NG25	6,26
	4,0 µM NG25	2,12
MM 2	0,0 µM NG25	1,61
	1,0 µM NG25	1,16
	2,0 µM NG25	0,76
MM 3	0,0 µM NG25	3,92
	2,0 µM NG25	3,08
	4,0 µM NG25	0,87
MM 4	0,0 µM NG25	36,7
	2,0 µM NG25	50,7
	4,0 µM NG25	43,4
MM 5	0,0 µM NG25	5,88
	2,0 µM NG25	7,98
	4,0 µM NG25	1,96
MM 6	0,0 µM NG25	2,78
	2,0 µM NG25	3,07
	4,0 µM NG25	0,07
MM 7	0,0 µM NG25	6,48
	6,0 µM NG25	4,72
	8,0 µM NG25	0,61
MM 8	0,0 µM NG25	2,83
	2,0 µM NG25	1,98
	4,0 µM NG25	0,85
MM 9	0,0 µM NG25	5,40
	6,0 µM NG25	1,28
	8,0 µM NG25	0,20
MM 10	0,0 µM NG25	4,14
	6,0 µM NG25	0,74
	8,0 µM NG25	0,001

$CD138^{+}$ cells from myeloma patients were treated with melphalan and the indicated doses of NG25 for 18 hours, and cell viability was determined by Cell Titer Viability assay. Results are average of technical triplicates for each donor.
